# Clinical Characteristics of Progressive Supranuclear Palsy Subtypes Defined by AT(N) CSF Biomarkers

**DOI:** 10.1002/alz.084808

**Published:** 2025-01-09

**Authors:** Takanobu Ishiguro, Kensaku Kasuga, Tamao Tsukie, Osamu Onodera, Takeshi Ikeuchi

**Affiliations:** ^1^ Department of Neurology, Brain Research Institute, Niigata University, Niigata Japan; ^2^ Department of Molecular Genetics, Brain Research Institute, Niigata University, Niigata, Niigata Japan; ^3^ Brain Research Institute, Niigata University, Niigata, Niigata Japan

## Abstract

**Background:**

Progressive supranuclear palsy (PSP) may show concomitant neuropathological findings such as Alzheimer's disease (AD)‐related pathologies. Recent advances in CSF biomarkers enabled the estimation of neuropathological changes in brains in various neurodegenerative diseases. Clinical characteristic of subtypes of PSP defined by CSF biomarkers have not been well understood.

**Methods:**

Nineteen PSP patients (11 males and 8 females, age at the examination: 72.9±7.9 years) who diagnosed by board‐certified neurologists were included in this study. They all met the criteria for 'probable,' 'possible,' or 'suggestive' PSP according to the MDS PSP criteria (2017). CSF AT(N) biomarkers including Aβ42 and Aβ42/40 ratio as A marker, p‐tau181 as T marker, and total tau [t‐tau] and neurofilament light chain [NfL]) as N marker were quantified. The positivity/negativity of each biomarker was determined based on the cutoff value defined in an independent cohort. PSP patients were classified into subgroup defined by AT(N) CSF biomarkers. Clinical characteristics were compared between the AD continuum (A+ profile) group and the non‐AD continuum (suspected non‐AD pathophysiology, SNAP) group. The activities of daily living (ADL) in PSP patients were evaluated using the Barthel Index.

**Results:**

Fourteen patients (74%) met the criteria for probable PSP‐Richardson syndrome. CSF AT(N) biomarker analysis revealed that the AD continuum pattern (A+ pattern) (AD [n=4] and, A+T‐N+ [n=8]) was observed in 12 PSP patients, and the SNAP pattern was observed in 7 PSP patients. The AD continuum group exhibited later onset (p=0.0009), lower MMSE scores (p=0.0177), and a higher frequency of axial rigidity (p=0.0379). CSF NfL showed a significant negative correlation with the Barthel Index in the entire PSP patients (r= ‐0.7281, p=0.001).

**Conclusions:**

Different clinical characteristics were observed in subgroups of PSP patients defined by AT(N) CSF biomarkers. PSP patients with the AD continuum had later onset and more impaired cognition than those without AD continuum. CSF NfL may be a valuable marker reflecting ADL in PSP patients.

